# Clinical correlation of cholelithiasis in patients undergoing percutaneous endoscopic gastrostomy

**DOI:** 10.1038/s41598-023-49417-2

**Published:** 2023-12-12

**Authors:** Boram Cha, Jungnam Lee, Jaehyuk Lee, Jin-Seok Park, Seok Jeong, Don Haeng Lee

**Affiliations:** 1 Department of Internal Medicine, Inha University Hospital, Inha University School of Medicine, Incheon, Republic of Korea; 2 Department of Internal Medicine, Shihwa medical center, Siheung, Republic of Korea

**Keywords:** Gastroenterology, Risk factors

## Abstract

The risk factor for cholelithiasis include low physical activity. With an aging society, the number of bedridden patients who undergo percutaneous endoscopic gastrostomy (PEG) has increased, and cholelithiasis has often been found in these patients. This study aimed to evaluate the risk factors correlated with cholelithiasis in adults who underwent PEG. This retrospective single-center design study reviewed patients who underwent PEG and were confirmed to have cholelithiasis through imaging from March 1996 to December 2021. The investigated variables were age, sex, body mass index (BMI, kg/m^2^), cause of PEG insertion, initial physical activity status, laboratory findings on PEG insertion day, and incidence of acute cholecystitis. The differences between categorical and continuous variables were analyzed using Student’s *t* test and *chi-*square test. We enrolled 576 eligible patients who underwent PEG insertion. A total of 161 patients were detected with cholelithiasis (28.0%). The overall independent risk factors for cholelithiasis in patients who underwent PEG insertion were increased C-reactive protein (CRP) levels and decreased physical activity status (bedridden state). The incidence of cholelithiasis was increased by up to 30.7%, especially in patients with bedridden status. However, the incidence of acute cholecystitis among cholelithiasis group was only 5.6%. BMI and total cholesterol were positively correlated with the size of gallbladder (GB) stones. One of the major risk factors for cholelithiasis is decreased physical activity, especially in patients who underwent PEG insertion. Abdominal imaging is recommended to confirm the presence of cholelithiasis and to consider prophylaxis for cholelithiasis, especially in bedridden patients with elevated initial CRP levels at the time of PEG insertion.

## Introduction

Cholelithiasis is a common disorder of the gastrointestinal system and frequent cause of hospitalization^1^. The estimated overall prevalence of the disease is 10–15% in the United States and Europe^1,2^. In South Korea, the prevalence of cholelithiasis ranges from 2 to 5%, and the number of patients who were diagnosed with cholelithiasis is increasing every year^3^. The clinical course of cholelithiasis ranges from the asymptomatic carriers to complicated symptomatic diseases, such as acute cholecystitis, cholangitis, common bile duct stones, or gallstone pancreatitis. Acute cholecystitis appears to occur in about 1–2% of asymptomatic patients with cholelithiasis annually. In patients with stones and mild symptoms, the rate of the development of acute cholecystitis increases by approximately 1–3% per year. The incidence of cholecystectomy due to severe symptoms is reported as being 6–8% per year^4^.

Cholelithiasis is caused by an interaction of genetic; environmental; metabolic, such as obesity, type 2 diabetes mellitus (DM), and dyslipidemia; and systemic, such as viral or bacterial infection and liver disease, risk factors^5,6^. Additionally, several studies have suggested that physical activity could be a protective factor and reduces the chance of developing cholelithiasis^7,8^.

Enteral nutrition (EN) involves the administration of a tailored liquid nutritional mixture to the specific needs of the patient by tube feeding into the stomach or small intestine^9,10^. Although most ENs begin with the insertion of a Levin tube (L-tube) into the nose for short-term purposes, if L-tube maintenance is required for more than 2–3 weeks, it is recommended to substitute percutaneous endoscopic gastrostomy (PEG) to prevent complications, including aspiration, esophageal bleeding, or esophageal structure^11–13^.

EN has been reported to be a potential risk factor for gallbladder (GB) dysfunction; however, the results were inconsistent and have not yet been clearly elucidated^14–16^. Continuous duodenal perfusion with liquid formulations has been shown to result in incomplete emptying of the GB, leading to sequestration of bile acids. Jawaheer et al. reported that continuous EN could impair GB contractility and lead to GB stasis^16^. However, another study showed that cholecystokinin concentrations remain elevated and GB was completely contracted during 6 weeks continuous administration of nutrients through EN. Therefore, this study suggests that long-term EN does not contribute to an increased prevalence of cholelithiasis^15^.

This study aimed to elucidate the factors contributing to the development of cholelithiasis, especially in patients with PEG insertion.

## Materials and methods

### Study design and participants

This study was conducted using a retrospective single-center tertiary design. A chart review was performed from March 1996 to December 2021 of patients who underwent PEG insertion and had or did not have cholelithiasis through imaging. The inclusion criteria were as follows: adults with PEG insertion whose cholelithiasis was or was not confirmed on abdominal imaging, including abdomen, chest computed tomography (CT), or ultrasonography. The exclusion criteria were as follows: < 18 years of age, history of cholecystectomy or cholelithiasis before starting L-tube feeding, and no imaging studies.

### Demographic and clinical variables

Patient demographic data at admission for PEG insertion included age; sex; body mass index (BMI, kg/m^2^); cause of PEG insertion, including aspiration due to old age or dementia, cerebral infarction or hemorrhage, lung disease, dysphagia due to oral or esophageal obstruction, others including neuromuscular diseases; and the presence of pre-existing diseases, such as hypertension (HTN), DM, congestive heart failure, cerebral infarction, myocardial infarction, coronary artery obstructive disease, or end-stage renal disease. Serum concentrations of lipid profiles, including total cholesterol, low-density lipoprotein-cholesterol, triglyceride, and hepatobiliary laboratory findings, including total bilirubin, aspartate aminotransferase, alanine aminotransferase, alkaline phosphatase, and C-reactive protein (CRP), were collected from the database within 1 month after PEG insertion. The physical activity level of the patients was analyzed to determine their fall risk at the time of admission through nurse records. Bedridden state was calculated “0,” able to walk with other’s help “1,” and walk by oneself voluntarily “2.”

### Clinical outcomes

The primary outcome was to determine the associated risk factors for cholelithiasis in patients who underwent PEG. The secondary outcome was the incidence of acute cholecystitis, size changes of GB stones or sludge in patients who underwent PEG insertion, and predictive factors in patients with increased GB stone size.

### Diagnosis of cholelithiasis

The diagnosis of cholelithiasis at any point during the period of EN with L-tube or PEG follow-up was recorded, along with abdominal imaging, including abdominal or chest CT, or abdominal ultrasonography. The CT and ultrasonography images were retrospectively reviewed from the records read by radiologists. The interval change in the GB stone size was determined by the width between the first detection and the last follow-up abdominal image.

### Statistical analysis

Categorical variables were presented as numbers (%) and numerical variables as means ± standard deviations or median with interquartile ranges. Numerical variables were analyzed using the independent-samples t-test, and categorical variables were analyzed using Fisher’s exact test. To determine the factors associated with cholelithiasis, univariate and multivariate analyses were performed using logistic regression models. The odds ratios (ORs) with 95% confidence intervals (CIs) were described. Covariates with a probability value *p* < 0.1 in the univariate analyses were included in the multivariate analysis. Statistical significance was set at *p* < 0.05.

### Ethics

The study protocol was approved by the Institutional Review Board (INHAUH 2022-02-002), and due to the retrospective nature of this study, the acquisition of informed consent from enrolled patients was waived. The study was conducted in accordance with the Declaration of Helsinki and Good Clinical Practice guidelines.

## Results

### Demographic and laboratory tests for the study population

**Figure 1:**
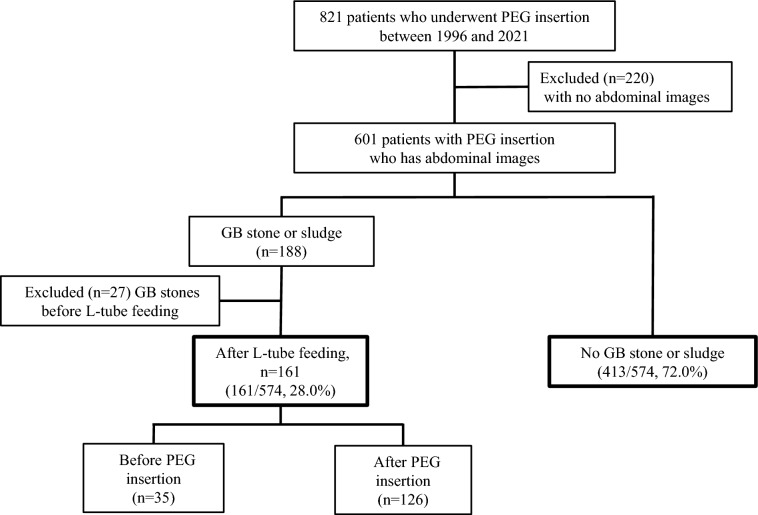
Flow diagram of patients with PEG insertion included in the study. PEG, percutaneous endoscopic gastrostomy; GB, gallbladder; L-tube, Levine-tube.

In Fig. 1, the medical records of 821 patients who underwent PEG insertion at our institution from March 1996 to December 2021 were analyzed (Fig. 1). A total of 220 patients were excluded due to a lack of imaging studies, and 601 patients were finally enrolled. A total of 188 patients had cholelithiasis; however, 27 patients were excluded because of cholelithiasis prior to L-tube feeding. Among 574 patients who started EN, 161 patients (161/574, 28%) had cholelithiasis, and 413 patients did not have cholelithiasis (413/574, 72%).

The patients’ mean age at the time of PEG insertion among adults was 68 ± 14 years, 71.1% were men, and the median BMI was 19.9 (9.6–31.6) kg/m^2^. Among the patients’ medical histories, 44.3%, 30.3%, and 27% patients had HTN, malignancy, and DM, respectively.

The most common cause for undergoing PEG insertion was brain damage (41.5%), including cerebral infarction, cerebral hemorrhage, or cerebral malignancy. The second most common cause was oropharyngeal or esophageal cancer obstruction (20.8%) and others, including neuromuscular disease (20.7%), which affect muscle movement or genetic diseases.

According to physical activity status, most patients for whom PEG insertion was indicated were in the bedridden state (462/574, 80.5%). Among voluntary walking patients, the majority were patients with oropharyngeal or esophageal obstruction (75/89, 84.3%) (Table 1).

**Table 1:** Basic and clinical characteristics of the patients with or without cholelithiasis who underwent PEG insertion.

	All (n = 574)	Non-cholelithiasis (n = 413)	Cholelithiasis (n = 161)	*p* value
Age (years, SD)	67.48 (14.28)	66.11 (14.26)	70.97 (13.79)	< 0.01
Sex (female), n (%)	164 (28.9)	116 (28.50)	48 (29.80)	0.756
BMI (kg/m^2^, SD)	19.89 (3.70)	19.70 (3.54)	20.26 (4.01)	0.154
PEG cause, n (%)				
Dementia, old age	75 (13.1)	33 (8)	42 (13.1)	
Brain damage	237 (41.5)	162 (39.5)	75 (46.6)	
Oropharyngeal or esophageal cancer or abnormality	119 (20.8)	94 (22.9)	25 (15.5)	
Lung disease	22 (3.9)	18 (4.4)	4 (2.5)	
Others	118 (20.7)	103 (25.1)	15 (9.3)	
Underlying disease, n (%)				
DM	153 (27)	100 (24.5)	53 (33.3)	0.03
HTN	251 (44.3)	177 (43.4)	74 (46.5)	0.496
ESRD	17 (3.0)	7 (1.7)	10 (6.3)	0.004
CAOD, CHF	44 (7.8)	33 (8.1)	11 (6.9)	0.629
LC	4 (0.7)	2 (0.5)	2 (1.3)	0.327
Malignancy	172 (30.3)	128 (31.4)	44 (27.3)	0.335
Laboratory findings, mean (median)				
WBC (×1000)	8.87 (5.01)	8.23 (3.83)	10.52 (6.97)	< 0.01
Hemoglobin (g/dL)	11.21 (2.14)	11.62 (6.70)	11.29 (2.36)	0.562
Total bilirubin (mg/dL)	0.77 (3.75)	0.75 (4.32)	0.81 (1.31)	0.865
AST (IU/L)	33.24 (52.254)	27.90 (22.52)	47.12 (90.95)	< 0.01
ALT (IU/L)	28.36 (46.48)	23.67(23.09)	40.55(78.84)	< 0.01
ALP (IU/L)	174.69 (195.79)	164 (163.47)	203.39 (262.22)	0.088
BUN (mg/dL)	20.949 (17.63)	18.49 (13.04)	27.57 (25.15)	< 0.01
Creatinine (mg/dL)	0.95 (0.96)	0.81 (0.73)	1.31 (1.33)	< 0.01
Total cholesterol (mg/dL)	140.15 (43.59)	142.44 (41.79)	134.42 (47.46)	0.063
LDL (mg/dL)	87.76 (47.79)	89.39 (47.82)	79.25 (47.76)	0.343
TG (mg/dL)	117.03 (68.70)	115.33 (64.21)	125.48 (88.78)	0.5
LDH (IU/L)	269.21 (193.21)	264.11 (201.64)	281.46 (171.39)	0.397
CRP (mg/dL)	4.61 (5.64)	3.71(4.36)	7.05(7.68)	< 0.01
Physical activity status (%)				< 0.001
Bed-ridden	462 (80.5)	320 (77.5)	142 (88.2)	
Involuntary walking	23 (4.0)	20 (4.8)	3 (1.9)	
Voluntary walking	89 (15.5)	73 (17.7)	16 (9.9)	

PEG, percutaneous endoscopic gastrostomy; n, number; BMI, body mass index; DM, diabetes mellitus; HTN, hypertension; ESRD, end stage renal disease; CAOD, coronary artery obstructive disease; CHF, congestive heart failure; LC, liver cirrhosis; WBC, white blood cell count; AST, aspartate aminotransferase; ALT, alanine aminotransferase; ALP, alkaline phosphatase; BUN, blood urea nitrogen; LDL, low density lipoprotein; TG, triglyceride; LDH, lactate dehydrogenase; CRP, C-reactive protein.

### Prevalence of cholelithiasis

The characteristics of the entire study population stratified by abdominal imaging diagnoses are detailed in Tables 1, 2, 3, 4, 5, 6. Patients were classified according to the occurrence of cholelithiasis into a cholelithiasis group (n = 161) and a non-cholelithiasis group (n = 413). Older age, DM, end stage renal disease (ESRD), elevated white blood cell count (WBC), aspartate transaminase (AST), alanine transaminase (ALT), blood urea nitrogen (BUN), creatinine, C-reactive protein (CRP), and physical activity status were significantly different between patients with PEG insertions and cholelithiasis and those without cholelithiasis (*p* < 0.05) (Table 1).

**Table 2:** Predictive factors associated with cholelithiasis in patients who underwent PEG insertion.

	*p* value	Odds ratio (95% CI)
WBC	0.109	1.039 (0.992–1.088)
AST	0.511	1.004 (0.993–1.014)
ALT	0.297	1.006 (0.995–1.017)
BUN	0.386	1.008 (0.990–1.025)
Creatinine	0.15	1.383 (0.890–2.149)
CRP	< 0.01	1.085 (1.040–1.132)
DM	0.846	1.051 (0.634–1.741)
ESRD	0.75	0.756 (0.135–4.224)
Bedridden state	< 0.01	3.174 (1.517–6.643)

PEG, percutaneous endoscopic gastrostomy; WBC, white blood cell count; AST, aspartate aminotransferase; ALT, alanine aminotransferase; BUN, blood urea nitrogen; CRP, C-reactive protein; DM, diabetes mellitus; ESRD, end stage renal disease.

**Table 3:** Predictive factors associated with cholelithiasis in patients who underwent PEG insertion, especially those who were bedridden.

	*p* value	Odds ratio (95% CI)
WBC	0.042	1.056 (1.002–1.113)
AST	0.425	1.005 (0.992–1.018)
ALT	0.441	1.005 (0.993–1.017)
BUN	0.293	1.01 (0.991–1.030)
Creatinine	0.352	1.239 (0.789–1.946)
CRP	< 0.01	1.096 (1.043–1.152)
ESRD	0.901	0.895 (0.155–5.181)

PEG, percutaneous endoscopic gastrostomy; WBC, white blood cell count; AST, aspartate aminotransferase; ALT, alanine aminotransferase; BUN, blood urea nitrogen; CRP, C-reactive protein; ESRD, end stage renal disease.

**Table 4:** Incidences of acute cholecystitis in patients who underwent PEG insertion.

Total incidence of acute cholecystitis	10/574 (1.7)
Cholelithiasis	9/161 (5.6%)
Median time interval since EN (min–max)	14 months (1–135)
No cholelithiasis	1/413 (0.2%)
Time interval since EN	100 months

PEG, percutaneous endoscopic gastrostomy; EN, enteral feeding.

**Table 5:** Changes in size of GB stone or sludge in patients who underwent PEG insertion.

GB stone or sludge follow-up image, n	n = 88
Size increased	22 (25.0%)
Increased size (min–max)	3.75 mm (0.6–15.0)
Monthly increase rate (min–max)	0.14 mm (0.02–1.0)
No size change	66 (75.0%)

GB, gallbladder; PEG, percutaneous endoscopic gastrostomy.

**Table 6:** Predictive factors associated with GB stone size increase in patients who underwent PEG insertion and had GB stone or sludge since L-tube feeding.

Variables	Univariate analysis		Multivariate analysis	
	OR (95% CI)	*p* value	OR (95% CI)	*p* value
Age	1.01 (0.97–1.04)	0.65		
Sex (male)	0.66 (0.23–1.89)	0.44		
BMI	1.13 (1.00–1.27)	0.04	1.15 (1.00–1.31)	0.04
PEG cause				
Dementia, old age	1.78 (0.45–6.94)	0.41		
Brain damage	5.06 (1.17–21.84)	0.03		
Oropharyngeal or esophageal cancer or abnormality	4.33 (0.34–55.48)	0.26		
Lung disease	2.0 (0.3–13.32)	0.47		
Laboratory findings				
WBC	1.01 (0.95–1.01)	0.69		
Hemoglobin	1.29 (1.04–1.60)	0.02		
Total bilirubin	0.80 (0.41–1.55)	0.51		
AST	0.98 (0.97–1.01)	0.27		
ALT	0.96 (0.99–1.01)	0.42		
ALP	0.99 (0.99–1.00)	0.29		
BUN	0.98 (0.93–1.03)	0.33		
Creatinine	0.99 (0.71–1.41)	0.99		
Total cholesterol	1.01 (1.00–1.02)	0.01	1.01 (1.00–1.02)	0.019
LDL	1.01 (0.99–1.04)	0.21		
TG	1.01 (0.99–1.02)	0.18		
LDH	0.99 (0.99–1.00)	0.22		
CRP	0.98 (0.91–1.05)	0.61		

GB, gallbladder; PEG, percutaneous endoscopic gastrostomy; L-tube, Levin-tube; n, number; BMI, body mass index; WBC, white blood cell count; AST, aspartate aminotransferase; ALT, alanine aminotransferase; ALP, alkaline phosphatase; BUN, blood urea nitrogen; LDL, low density lipoprotein; TG, triglyceride; LDH, lactate dehydrogenase; CRP, C-reactive protein.

According to physical activity status, bedridden patients had significantly higher proportion in the cholelithiasis group than that in the non-cholelithiasis group (“0,” 88.2% vs. 77.5%). In contrast, increased physical activity status both in involuntary (“1,” 1.9% vs. 4.8%) and voluntary (“2,” 9.9% vs. 17.7%) showed significantly low proportions of cholelithiasis (*p* < 0.001).

### Risk factors for cholelithiasis in patients who underwent PEG insertion

According to the Cox proportional hazard model analyses, the independent predictors for cholelithiasis in the overall population of patients who underwent PEG insertion are shown in Table 2. Only CRP (OR, 1.09; 95% CI 1.04–1.31, *p* < 0.01) and bedridden state of physical activity variables (OR, 3.17; 95% CI 1.52–6.64, *p* < 0.01) were found to have significant predictive effects.

### Predictive factors associated with cholelithiasis in patients who underwent PEG insertion

Due to the high correlation between the bedridden state and cholelithiasis in patients who underwent PEG insertion, we selectively performed Cox proportional hazard model analyses to determine the independent predictors for cholelithiasis (Table 3). WBC (OR, 1.06; 95% CI 1.00–1.11; *p* = 0.04) and CRP (OR, 1.10; 95% CI 1.04–1.15; *p* < 0.01) variables were found to have significant predictive effects.

### Incidence of acute cholecystitis among patients who underwent PEG insertion

The incidence of acute cholecystitis in patients who underwent PEG insertion is shown in Table 4. Among 574 patients who underwent PEG insertion, 10 patients experienced symptomatic cholecystitis (10/574, 1.74%), 9 (9/161, 5.6%) patients developed calculous cholecystitis at a median of 14 months (1–135) after L-tube feeding, and only one patient developed calculous cholecystitis from the non-cholelithiasis group (1/10, 10%) at 100 months after L-tube feeding. Acute cholecystitis was diagnosed incidentally in all 10 patients with sustained fever by imaging tests performed in the emergency department, and six patients underwent percutaneous transhepatic GB drainage instead of surgical resection because of old age or high risk of general anesthesia. The rest 152 patients (98.3%) were silent cholelithiasis patients.

### Changes in size of GB stone or sludge in patients who underwent PEG insertion

Although the majority of patients with cholelithiasis who underwent PEG were silent (98.3%), 23.7% showed an increase in GB stones or sludge size (Table 5) over time. The median increase in size was 3.75 mm (0.6–15.0), and the median monthly increase in size was 0.14 mm (0.02–1.0). However, the majority of GB stones showed no interval size change (66/88, 75%). Table 6 shows the univariate and multivariate analyses of the factors affecting the increased risk of GB stone size. In the univariate analyses, BMI, brain damage, hemoglobin, and total cholesterol were positively and significantly associated with increased GB stone size. In the multivariate analyses, BMI (OR, 1.15; 95% CI 1.00–1.31, *p* = 0.04) and cholesterol levels (OR, 1.01; 95% CI 1.00–1.02, *p* = 0.019) were independently associated with increased GB stone size.

## Discussion

To the best of our knowledge, this is the first study to explore the incidence, risk factors, and clinical outcomes of cholelithiasis in patients who underwent PEG insertion. The incidence of cholelithiasis in adults who underwent PEG insertion was 28%, and the incidence of symptomatic acute cholecystitis was 1.7% in the PEG insertion group; 5.6% in the cholelithiasis group and 0.2% in the non-cholelithiasis group. Moreover, we found that decreased physical activity was a crucial independent risk factor for cholelithiasis in patients who underwent PEG insertion.

EN has been reported to be one of potential risk factor for GB dysfunction; however, the results were inconsistent and remain controversial^14,16^. However, these studies included patients with different characteristics compared to our cohort. Due to the retrospective nature of our study, we did not evaluate GB dysfunction. Still, our study revealed that 28% of the patients who underwent PEG insertion developed cholelithiasis. This incidence is strikingly higher than the 2–5% overall rate of cholelithiasis reported in South Korea^3^. However, because of their characteristics, patients who undergo PEG insertion are already exposed to various factors that affect cholelithiasis; therefore, we could not conclude that the incidence of cholelithiasis is increased solely by EN.

We evaluated well-known risk factors for cholelithiasis in patients who underwent PEG insertion. Risk factors for cholelithiasis in the general population include DM, BMI, TG, female sex, age, and physical activity^17–20^. Decreased physical activity status was the most common independent risk factor for cholelithiasis in patients who underwent PEG insertion. In addition, increased CRP levels were found to be an independent risk factor. Among these factors, decreased physical activity status showed the highest positive correlation with an OR of 3.17 (*p* < 0.01). As a result, the overall incidence of cholelithiasis increased from 28.0% (161/574) to 30.7% (142/462) when we assessed separately in a bedridden state, confirming that physical activity plays the most important role in cholelithiasis.

Regarding the distribution of patients who underwent PEG insertion, 81% of them were in the bedridden state due to brain damage or neuromuscular disorders. To determine the cause of cholelithiasis risk in the most patients who underwent PEG insertion, we separately evaluated risk factors in their bedridden state (Table 3). The only common independent variable was CRP (OR, 1.01); however, its correlation was not as high as that of physical activity (OR, 3.17). This finding reinforces the importance of physical activity in cholelithiasis.

In this study, we investigated the association between EN and cholelithiasis development (Table 5) and determined the risk factors (Table 6) for GB stone size increase in patients who underwent PEG insertion. Only 88 patients underwent follow-up CT or ultrasonography. Among them, the GB stones in 25% (22/88) of patients increased in size, and the median monthly increase rate was 0.14 mm with high total cholesterol level, and BMI was positively correlated with GB stone size increase.

In addition, 10 patients (1.7%) developed acute cholecystitis requiring surgical or trans-hepatic GB drainage in our study. Calculous cholecystitis was found in 5.6% of them, which is a relatively modest percentage compared with markedly increased incidence of cholelithiasis in patients who underwent PEG insertion. In other words, there are relatively more silent GB stone proportions in patients who underwent PEG insertion than those in the general population. Nevertheless, as a significant number of patients who underwent PEG insertion have underlying dementia or brain disease, even if acute cholecystitis occurs, they are often unable to self-report the typical symptoms that clinicians suspect of cholecystitis and can become critically ill, even causing sepsis and death. Therefore, clinicians should be cautious of patients with PEG insertion in bedridden states, especially those with cholelithiasis. Moreover, acute cholecystitis should be suspected if leukocytosis with neutrophilia, serum total bilirubin, and alkaline phosphatase concentrations are found.

Due to the many predisposing factors for cholelithiasis in patients who underwent PEG insertion, there is limited evidence that PEG or EN itself increases the incidence of cholelithiasis. However, even after excluding the most important factor, which is reduced physical activity, the incidence of cholelithiasis in patients who underwent PEG insertion was 18.4%, which is still much higher than that of the general population. Although the mechanism underlying the correlation between PEG and cholelithiasis needs to be investigated, our study can conclude that PEG itself affects GB dyskinesia.

The limitations of this study are as follows. First, this was a retrospective, single-institution study conducted at a tertiary hospital center. Given this retrospective design, we recognize that CT might have a lower sensitivity in detecting cholelithiasis, especially cholesterol stones, compared to US. This might have led to some missed detections, particularly of cholesterol stones which are not as readily visible on CT as other types. Furthermore, variations in the sensitivity and specificity of detection between dynamic CT and US across different studies are acknowledged, and this heterogeneity could influence our results. Second, as the time of occurrence of cholelithiasis is unknown, prolonged EN cannot explain the increased occurrence of cholelithiasis. Third, the absolute risk of cholelithiasis attributable to PEG remains unknown because of the lack of a control group of patients without PEG. Fourth, the exact Eastern Cooperative Oncology Group (ECOG) status of the patients could not be evaluated. However, we tried to evaluate nurse records from fall risk at the time of admission for PEG insertion. In the future, prospective studies are required to further investigate these unresolved issues. However, to the best of our knowledge, this is the first study to find a higher incidence of cholelithiasis in patients with PEG insertion.

In conclusion, adult patients with PEG insertion, especially those in the bedridden state, are at a particularly high risk of developing cholelithiasis. Although the incidence of acute cholecystitis in patients with PEG insertion is relatively low, the risk of serious complications may be high because patients cannot express symptoms. Therefore, initial abdominal imaging studies should be considered for high-risk patients at the time of PEG insertion, and the prophylactic administration of ursodeoxycholic acid may be beneficial in patients with GB stones.

## Data Availability

The datasets used and analysed during the current study available from the corresponding author on reasonable request.

## Abbreviations

PEG: Percutaneous endoscopic gastrostomy
GB: Gallbladder
EN: Enteral feeding
L-tube: Levin-tube
BMI: Body mass index
HTN: Hypertension
DM: Diabetes mellitus
COPD: Chronic destructive pulmonary disease
CHF: Congestive heart failure
MI: Myocardial infarction
CAOD: Coronary artery obstructive disease
ESRD: End-stage renal disease
